# The relationship between the hierarchical position of proteins in the human signal transduction network and their rate of evolution

**DOI:** 10.1186/1471-2148-12-192

**Published:** 2012-09-28

**Authors:** David Alvarez-Ponce

**Affiliations:** 1 Department of Biology, National University of Ireland Maynooth, Maynooth, County Kildare, Ireland; 2 Instituto de Biología Molecular y Celular de Plantas, Consejo Superior de Investigaciones Científicas (CSIC)-Universidad Politécnica de Valencia (UPV), Valencia, Spain

**Keywords:** Network evolution, Evolutionary rate, Selective constraint, Purifying selection, Signal transduction, Molecular pathways, Upstream and downstream position

## Abstract

**Background:**

Proteins evolve at disparate rates, as a result of the action of different types and strengths of evolutionary forces. An open question in evolutionary biology is what factors are responsible for this variability. In general, proteins whose function has a great impact on organisms’ fitness are expected to evolve under stronger selective pressures. In biosynthetic pathways, upstream genes usually evolve under higher levels of selective constraint than those acting at the downstream part, as a result of their higher hierarchical position. Similar observations have been made in transcriptional regulatory networks, whose upstream elements appear to be more essential and subject to selection. Less well understood is, however, how selective pressures distribute along signal transduction pathways.

**Results:**

Here, I combine comparative genomics and directed protein interaction data to study the distribution of evolutionary forces across the human signal transduction network. Surprisingly, no evidence was found for higher levels of selective constraint at the upstream network genes (those occupying more hierarchical positions). On the contrary, purifying selection was found to act more strongly on genes acting at the downstream part of the network, which seems to be due to downstream genes being more highly and broadly expressed, performing certain functions and, in particular, encoding proteins that are more highly connected in the protein–protein interaction network. When the effect of these confounding factors is discounted, upstream and downstream genes evolve at similar rates. The trends found in the overall signaling network are exemplified by analysis of the distribution of purifying selection along the mammalian Ras signaling pathway, showing that upstream and downstream genes evolve at similar rates.

**Conclusions:**

These results indicate that the upstream/downstream position of proteins in the signal transduction network has, in general, no direct effect on their rates of evolution, suggesting that upstream and downstream genes are similarly important for the function of the network. This implies that natural selection differently distributes across signal transduction networks and across biosynthetic and transcriptional regulatory networks, which might reflect fundamental differences in their function and organization.

## Background

Proteins’ rates of evolution vary across orders of magnitude (e.g., refs. [1,2]). Understanding the factors underlying this enormous variability is a central problem in evolutionary biology. The neutral theory of molecular evolution predicts higher selective pressures (and hence, slower rates of evolution) acting on genes encoding proteins that perform the most important functions for organisms’ biological fitness [3]; however, the relative contributions of genes to fitness remain elusive. Over the last decade, the availability of both genomic and functional data has allowed us to identify some of the factors that correlate with genes’ rates of evolution, among which patterns and levels of gene expression appear to be the most important (for review, see refs. [4-6]). However, this gene-centered approach has been able to explain only a relatively small fraction of the variability of rates of evolution (e.g., ref. [7]).

Genes and proteins do not act in isolation, but usually operate as components of complex networks of interacting molecules. Therefore, taking into account the interaction networks in which a gene participates may provide key insight into the evolutionary forces acting on it. Furthermore, understanding the function and evolution of molecular networks can aid applications such as metabolic engineering and drug discovery and design (for review, see refs. [8-10]). Molecular networks in general can be modeled as undirected graphs, whose nodes and edges represent proteins and interactions, respectively. Over the last years, the accumulation of interactomic data has allowed us to cast a first glance at the impact of genes’ position in the network on their patterns of molecular evolution (reviewed in refs. [11-13]). For instance, genes encoding the most connected proteins in metabolic and protein–protein interaction networks are more selectively constrained than those acting at the network periphery [14-19], and interacting genes tend to show correlated evolutionary histories, e.g. evolving at similar rates [14,16,20-24]. These observations indicate that the structure of molecular networks impose constraints on the evolution of their components.

In addition to the centrality of proteins in functional networks, their hierarchical position in molecular pathways has also been put forward as a determinant of their rates of evolution. Remarkably, in a number of biosynthetic pathways, the strength of purifying selection has been found to correlate with the position of genes along the upstream/downstream pathway axis, with upstream genes being generally the most constrained [25-30]. This distribution of the levels of selective constraint has been attributed to upstream genes being involved in the biosynthesis of a broader range of biochemical compounds than downstream genes, and hence being more pleiotropic [28], and/or to upstream genes exerting a higher influence over the flux of metabolites along pathways [31]. Consistent observations have been made in transcriptional regulatory networks: upstream genes in these networks are more likely to be essential [32], and simulation analysis has shown that genes exerting a higher degree of control over other genes, and/or those that are less regulated by other genes, are more strongly affected by selection [33].

Less well understood is how selective pressures distribute along signal transduction pathways. These pathways play a key role in modulating cell function in response to extracellular and intracellular stimuli. They are characterized by the presence of a receptor that, in response to signals, is able to trigger a cascade of events that ultimately modulate the final effectors, which are responsible for mediating the biological responses of the pathway. The signal is transmitted from the upstream to the downstream part of the pathway by means of a series of molecular interactions (e.g., phosphorylation and/or dephosphorylation events). These interactions have a directed nature, i.e., each interaction involves an upstream modulator (either activator or inhibitor) and a downstream modulated element (activated or inhibited). Therefore, signaling pathways and networks can be represented as directed graphs, in which nodes and arcs represent molecules and directed interactions, respectively. Despite the availability of some directed interaction networks (e.g., refs. [34-36]), their directed nature has often been overlooked in evolutionary analyses, being often treated as undirected networks.

So far, the distribution of levels of selective constraint across the upstream/downstream axis of signaling pathways has been studied in only a few pathways, with contrasting results. In the *Drosophila* Ras pathway, upstream genes appear to be more selectively constrained than those acting at the downstream part [37]. This is to be expected under a model in which upstream genes, owing to their higher hierarchical position, have a higher impact on the function of the pathway. Indeed, a single activated upstream protein has the potential to activate several downstream target molecules (e.g., ref. [38]); therefore, higher levels of selective constraint might be expected at these genes. However, the opposite pattern (i.e., stronger purifying selection acting on downstream genes) has been described in the *Drosophila*, *Caenorhabditis* and vertebrate insulin/TOR pathway [39-42], and in the yeast HOG pathway [43]. In both the yeast HOG pathway and the *Caenorhabditis* insulin/TOR pathway, the tendency appears to vanish when the effect of confounding factors that correlate with both network position and levels of selective constraint, such as expression level or codon bias, are removed [42,43], whereas in the *Drosophila* and vertebrate insulin/TOR pathway the polarity in the levels of selective constraint is independent of these and other factors [39-41,44]. Therefore, the effect of upstream/downstream position of genes in signal transduction pathways on their rates of evolution, and the relative contribution of potential confounding factors, remain elusive.

Here, I combine comparative genomics and manually-curated directed protein interaction data to determine whether purifying selection differentially acts on the upstream and downstream genes of the human signal transduction network. For that purpose, new methods that explicitly take into account the directed character of the network are introduced. Surprisingly, no evidence was found supporting that upstream genes (those occupying higher hierarchical positions) are subject to increased levels of selective constraint. On the contrary, purifying selection was found to act more severely on genes acting at the downstream part of the network, which seems to be due to downstream genes being more highly and broadly expressed, having certain functions and, in particular, encoding proteins that are more highly connected in the protein–protein interaction network. When the effect of these confounding factors was discounted, upstream and downstream genes were found to evolve at similar rates. These results clearly indicate that the hierarchical position of genes in the signal transduction network has, in general, no direct effect on their rates of evolution, suggesting that genes occupying more hierarchical positions in the network are not more relevant for the function of the network and the fitness of the organism. The trend found in the overall signaling network is exemplified by analysis of the distribution of purifying selection along the mammalian Ras signaling pathway, showing that, contrary to previously suggested in *Drosophila*, upstream and downstream genes evolve at similar rates. These observations imply that natural selection differently distributes across signal transduction networks and across biosynthetic and transcriptional regulatory networks. This might reflect fundamental differences in the function and organization of the signal transduction network and metabolic and transcriptional regulatory networks.

## Results

### Unexpectedly, genes acting at the upstream part of the human signal transduction network exhibit higher rates of evolution

The human signal transduction network gleaned by Cui *et al*. [34] was used. In this network, directed edges (links) between pairs of proteins represent regulatory interactions (i.e., activating or inhibitory) — e.g., the directed edge “A → B” denotes that protein “A” activates or inhibits protein “B”. The dataset was assembled and manually curated by Cui *et al*. by merging the interactions compiled in different databases, followed by validation of each interaction by accurate revision of the literature. Undirected edges, which represent generic (i.e., non-signaling) physical interactions, were not considered in the current analysis, since they contain no information about the hierarchical (upstream/downstream) position of genes in the network. After filtering the dataset (see Methods), it comprised 1049 proteins connected by 2436 non-redundant directed interactions (activating or inhibitory). This dataset included 10 ligands, 102 receptors, 62 adapters, 187 kinases, 28 phosphatases, 123 transcription factors, 99 structural proteins, and 27 ion transmembrane transporters. Most edges represent direct physical protein–protein interactions, with the only exception of a small fraction of “transcription factor → target gene” edges.

For each gene in the network, the 1:1 mouse ortholog was identified and the impact of natural selection was characterized from the nonsynonymous to synonymous divergence ratio (*ω* = *d*_N_/*d*_S_). Values of *ω* equal to 1 are expected for genes evolving neutrally, whereas *ω* < 1 is indicative of the action of purifying selection preserving the sequence of the encoded proteins, and *ω* > 1 in a number of codons is indicative of the action of positive selection (adaptive evolution) driving the fixation of nonsynonymous substitutions. The estimated *ω* values range from 0.0001 to 0.814 (with a median value of 0.065), indicating that purifying selection has played an important role in the evolution of these genes.

I evaluated whether *ω* values depend on the hierarchical position that genes occupy in the network. For that purpose, three different approaches were adopted to compare the evolutionary rates of upstream and downstream network genes. First, the *ω* value of each gene was compared with the central value of the *ω* values of all its direct downstream targets (the median in the case of an odd number, or one of the two central values in the case of an even number of downstream proteins; see Methods). Out of the 1049 genes in the network, 800 encode proteins that have at least one direct downstream target in the network (i.e., out-degree > 0). Of those, 446 genes exhibit a higher *ω* value than their targets, whereas 352 show lower *ω* values than their targets. This asymmetry is not compatible with a random distribution of *ω* values across upstream and downstream genes (paired sign test, *P* = 0.001; Table 1). Therefore, surprisingly, upstream genes tend to show higher *ω* values than the genes that they modulate. In order to establish whether the evolution of nonsynonymous or synonymous sites is responsible for this trend, this analysis was conducted for *d*_N_ and *d*_S_ separately. Clearly significant differences were observed for *d*_N_ (*P* = 3.50 × 10^–4^), but only marginally significant differences were observed for *d*_S_ (*P* = 0.040; Table 1), indicating that the evolutionary patterns of nonsynonymous sites are the main responsible for the trend observed in *ω*.

**Table 1:** **Paired tests comparing upstreamgenes with their downstreamtargets**

**Parameter**	***n***^**a**^	**Upstream > Downstream**^**b**^	**Upstream < Downstream**^**c**^	***P***
*ω*	800	446	352	0.001***
*d*_N_	800	450	348	3.50×10^–4^***
*d*_S_	800	429	370	0.040*
Expression level	763	357	406	0.082
Expression breadth	763	261	337	0.002**
ENC	800	398	402	0.916
Connectivity	709	230	456	8.66×10^–18^***
Number of paralogs	800	369	373	0.912

*, *P*<0.05; **, *P*<0.01, ***, *P*<0.001.

^a^Number of genes with at least one direct downstream target (out-degree>0). Only genes with available information for the parameter of interest were considered.

^b^Number of genes with a higher value than the central value of its downstream targets.

^c^Number of genes with a lower value than the central value of its downstream targets.

**Figure 1:**
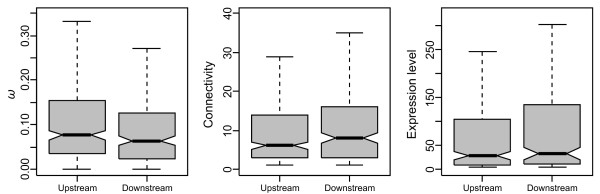
**Comparison of genes encodingthe upstream and downstreamproteins of the humansignal transduction network.**

Next, evolutionary rates of genes encoding proteins occupying extreme upstream and downstream positions in the network were compared. A total of 333 genes were classified as upstream [as they modulate other proteins (out-degree > 0) but are not modulated by any other protein in the network (in-degree = 0)] and 249 were classified as downstream [modulated by at least one protein (in-degree > 0), but unable to modulate any other protein (out-degree = 0)]. Again, upstream genes were found to exhibit significantly higher *ω* values: upstream and downstream genes exhibit median *ω* values of 0.076 and 0.064, respectively (Mann–Whitney test, *P* = 0.037; Figure 1). Significant differences were also observed in the *d*_N_ (*P* = 0.049), but not in the *d*_S_ (*P* = 0.782; Table 2) values.

**Table 2:** **Comparison of genes occupyingextreme upstream and downstreampositions in the signalingnetwork**

**Parameter**	**Upstream**			**Downstream**			***P***
	***n***	**Median**	**Average**	***n***	**Median**	**Average**	
*ω*	333	0.076	0.112	249	0.064	0.098	0.037*
*d*_N_	333	0.045	0.070	249	0.037	0.060	0.049*
*d*_S_	333	0.561	0.630	249	0.567	0.626	0.782
Expression level	322	27.32	116.28	243	32.43	165.13	0.239
Expression breadth	322	21.00	15.41	243	23.00	15.84	0.621
ENC	333	50.43	49.36	249	50.36	49.39	0.712
Connectivity	293	6.00	11.86	225	8.00	13.41	0.114
Number of paralogs	333	22.00	36.73	249	28.00	37.63	0.714

*P*-values were obtained from the Mann–Whitney *U* test. *, *P* < 0.05.

Finally, I evaluated whether *ω* values correlate with parameters that reflect the hierarchical position of genes in the network. Both in-degree (the number of upstream modulators or upcoming edges) and out-degree (number of downstream targets or outgoing edges) negatively correlate with *ω* values, consistent with previous observations that highly connected genes are subject to stronger purifying selection [14-16]. Remarkably, the correlation is stronger for in-degree (Spearman’s rank correlation coefficient, *ρ* = −0.116, *P* = 1.74 × 10^–4^) than for out-degree (*ρ* = −0.070, *P* = 0.023), indicating that the *ω* value of a gene is more influenced by the number of upstream genes (i.e., by how regulated the encoded product is), than by the number of downstream targets (i.e., by the level of regulation that it exerts on the network). Similar results were obtained for *d*_N_, whereas *d*_S_ does not correlate with in-degree or out-degree (Table 3). Beyond considering the direct downstream targets of a gene, I also considered the effect of the total number of genes acting directly or indirectly downstream of a given gene (termed *H*; see Methods) on its evolutionary rate. This parameter positively correlates with *ω* (*ρ* = 0.067, *P* = 0.031), again indicating that proteins potentially affecting the activity of a higher number of other proteins (i.e., those occupying higher hierarchical positions in the network) are encoded by genes that exhibit higher *ω* values. Again, significant results were obtained for *d*_N_ (*ρ* = 0.067, *P* = 0.030), but not for *d*_S_ (*ρ* = 0.016, *P* = 0.596; Table 3).

**Table 3:** **Bivariate correlations between parametersof interest and measuresof hierarchical positions ofgenes in the network**

** Variable 1**	**Variable 2**	***n***	***ρ***	***P***
*ω*	in-degree	1049	−0.116	1.74×10^–4^***
	out-degree	1049	−0.070	0.023*
	*H*	1049	0.067	0.031*
*d*_N_	in-degree	1049	−0.117	1.44×10^–4^***
	out-degree	1049	−0.072	0.019*
	*H*	1049	0.067	0.030*
*d*_S_	in-degree	1049	−0.035	0.259
	out-degree	1049	−0.014	0.653
	*H*	1049	0.016	0.596
Expression level	in-degree	1016	0.019	0.553
	out-degree	1016	−0.035	0.258
	*H*	1016	−0.033	0.293
Expression breadth	in-degree	1016	0.054	0.084
	out-degree	1016	0.044	0.164
	*H*	1016	−0.039	0.218
ENC	in-degree	1049	0.012	0.705
	out-degree	1049	−0.022	0.480
	*H*	1049	−0.024	0.437
Connectivity	in-degree	951	0.275	5.07×10^–18^***
	out-degree	951	0.230	7.38×10^–13^***
	*H*	951	−0.016	0.621
Number of paralogs	in-degree	1049	0.023	0.466
	out-degree	1049	0.047	0.131
	*H*	1049	0.023	0.451

*, *P* < 0.05; ***, *P* < 0.001.

Taken together, these results show that evolutionary rates exhibit a polarity across the human signaling network, with downstream genes progressively exhibiting lower *ω* and *d*_N_ values. This observation is in sharp contrast with patterns observed in metabolic and transcriptional regulatory pathways, in which upstream genes are generally the ones exhibiting lower rates of evolution [25-30,32,33]. I next considered a number of potential factors that could account for the observed polarity.

### The higher rates of evolution of upstream genes are the result of lower levels of purifying selection rather than a higher incidence of positive selection

The lower *ω* and *d*_N_ values observed in the downstream network genes suggest a higher strength of purifying selection acting on these genes. However, *ω* and *d*_N_ values are also affected by the action of positive selection, and hence the observed trends could alternatively be the result of a putatively higher incidence of positive selection on upstream genes. In order to discard this possibility, analyses were repeated after eliminating genes with the signature of positive selection.

When applied to pairs of human-mouse orthologs, the M7 *vs.* M8 test [45] identified 58 network genes with potential signatures of positive selection (i.e., a fraction of codons with *ω* > 1). Excluding these genes yielded similar results. First, after excluding these genes, 741 genes have at least one direct downstream target in the network, out of which 417 and 322 show higher and lower *ω* values than their targets, respectively (paired test, *P* = 0.001). Significant results were also obtained for *d*_N_ (*P* = 3.79 × 10^–5^). Second, the 315 genes classified as upstream that display no signals of positive selection exhibit significantly higher *ω* (Mann–Whitney test, *P* = 0.033), and *d*_N_ values (*P* = 0.049) than the 230 downstream genes without the signature of positive selection. Finally, *ω* and *d*_N_ values positively correlate with *H* (*ρ* = 0.065, *P* = 0.040 for *ω*; *ρ* = 0.064, *P* = 0.044 for *d*_N_), and more strongly correlate with in-degree (*ρ* = −0.115, *P* = 2.71 × 10^–4^ for *ω*; *ρ* = −0.115, *P* = 2.94 × 10^–4^ for *d*_N_) than with out-degree (*ρ* = −0.070, *P* = 0.028 for *ω*; *ρ* = −0.072, *P* = 0.023 for *d*_N_).

Because detection of positive selection is sensitive to the number of sequences used [46], analyses were repeated using a total of 8 mammalian genomes (human, chimpanzee, gorilla, orangutan, macaque, marmoset, mouse and rat). A total of 808 network genes exhibit a 1:1 orthology across all genomes, out of which 115 exhibit potential signals of positive selection. Again, excluding these genes did not alter the observations that genes acting at the downstream part of the network exhibit significantly lower *ω* (paired test, *P* = 1.05 × 10^–5^; Mann–Whitney test, *P* = 0.033; correlation with *H*, *ρ* = 0.065, *P* = 0.040) and *d*_N_ values (paired test, *P* = 0.001; Mann–Whitney test, *P* = 0.020; correlation with *H*, *ρ* = 0.072, *P* = 0.028).

Therefore, the distribution of *ω* and *d*_N_ values across the network may be the result of a polarity in the strength of purifying selection, with downstream genes being subject to higher levels of selective constraint, and not the result of a higher incidence of positive selection in the upstream part of the network.

### Subcellular location does not account for the higher rates of evolution of upstream network proteins

Genes acting at the extracellular compartment have been reported to exhibit high rates of evolution [20,47]. Consistently, among genes in the used dataset, those acting at the extracellular space exhibit higher *ω* and *d*_N_ values than the rest of the genes in the signal transduction network (Mann–Whitney test, *P* = 1.56 × 10^–16^ for *ω*; *P* = 2.23 × 10^–19^ for *d*_N_). This could potentially explain the observed polarity in *ω* and *d*_N_ values if proteins occupying highly hierarchical positions in the signaling network preferentially located to the extracellular space. However, I tested this possibility by comparing the proportion of extracellular proteins among proteins acting at the upstream (14.41%) and downstream (10.44%) parts of the network, finding no significant differences (Fisher’s exact test, *P* = 0.168). I.e., proteins occupying extreme upstream positions in the network do not tend to act at the extracellular space, thus making it unlikely that the observed high *ω* values in genes encoding the upstream part of the network would be linked to subcellular location. In order to further discard this possibility, analyses were repeated eliminating the 105 proteins targeted to the extracellular space, with similar results. First, genes are significantly less selectively constrained than their downstream direct targets (paired test, *P* = 0.001 for *ω*; *P* = 0.007 for *d*_N_). Second, the 285 non-extracellular proteins classified as upstream exhibit higher *ω* and *d*_N_ values than the 223 non-extracellular proteins acting at the downstream part of the network, although the differences are not statistically significant (median *ω* for upstream and downstream genes: 0.068 and 0.059, respecively; Mann–Whitney test, *P* = 0.110; *P* = 0.151 for *d*_N_). Third, the *ω* and *d*_N_ values correlate better with in-degree (*ρ* = −0.092, *P* = 0.005 for *ω*; *ρ* = −0.091, *P* = 0.005 for *d*_N_) than with out-degree (*ρ* = −0.051, *P* = 0.114 for *ω*; *ρ* = −0.054, *P* = 0.100 for *d*_N_). Although evolutionary rates do not significantly correlate with *H*, the direction of the correlations is positive and comparable in strength to that observed in the overall dataset (*ρ* = 0.052, *P* = 0.113 for *ω*; *ρ* = 0.053, *P* = 0.102 for *d*_N_). These results indicate that the polarity observed in the levels of selective constraint is for the most part independent of the high evolutionary rates of proteins acting at the extracellular space.

Using an expanded dataset (which included also generic physical, undirected interactions), Cui, Purisima and Wang [20] found that signaling genes acting at the cell membrane, cytoplasm and nucleus exhibited different rates of evolution. Again, these differences raise the possibility that the observed polarity in the *ω* values across the network might be in part the result of the distribution of upstream and downstream proteins across the different subcellular compartments. However, I found that (1) genes acting at the different cell compartments do not exhibit significantly different rates of evolution for the subset of genes included in the current analysis (which contained only genes involved in directed signaling interactions) (median *ω* values of 0.052, 0.056 and 0.064, respectively; Kruskal-Wallis test, *P* = 0.804), and that (2) the upstream and downstream parts of the network do not contain different proportions of proteins locating to the cell membrane (Fisher’s exact test, *P* = 0.738), cytoplasm (*P* = 0.773) or nucleus (*P* = 0.760), making it unlikely that the polarity in the levels of selective constraint described here would obey to proteins’ subcellular location. In order to further discard this possibility, analyses were conducted separately on subsets of genes acting at the different cell locations, with consistent results across all four compartments. First, genes tend to evolve faster than their direct downstream targets acting at the same cell compartment, when the analyses are restricted to genes acting at the extracellular space (15 and 10 proteins exhibit higher and lower *ω* values, respectively, than their downstream targets acting in the same compartment; paired test, *P* = 0.424), the cell membrane (126 and 88 proteins; *P* = 0.011), the cytoplasm (186 *vs.* 177; *P* = 0.675), or the nucleus (142 *vs.* 138; *P* = 0.858). Second, *ω* values are higher for upstream than for downstream genes when genes acting at the extracellular space (median values of 0.164 and 0.158 for upstream and downstream genes, respectively), the cell membrane (0.075 *vs.* 0.063), the cytoplasm (0.065 *vs.* 0.056) or the nucleus (0.0653 *vs.* 0.0646) are analyzed separately, although the differences are not statistically significant (Mann–Whitney test, *P* > 0.05). Taken together, these results indicate that the polarity in the levels of selective constraint observed across the network is independent of the subcellular location of its components. The lack of significance in some tests probably owes to the reduced statistical power resulting from partitioning the dataset.

### Gene functions upstream and downstream of the signal transduction network

Genes with different functions are known to evolve at different rates (e.g., refs. [23] and [48]). Hence, in order to gain further insight into the different selective pressures acting at the upstream and downstream parts of the human signaling network, it was tested whether the 333 genes acting at the upstream part performed different functions to the 249 genes acting at the downstream part. For that purpose, the frequencies of the GOslim GOA terms of both groups was compared using the FatiGO software [49]. Upstream genes exhibited a significant enrichment in the term “kinase activity” (*q* = 2.24 × 10^–4^), whereas downstream genes were enriched in the category “ion transmembrane transporter activity” (*q* = 2.65 × 10^–5^). Hence, the higher levels of selective constraint observed in the downstream part of the signaling network could potentially result from this unequal distribution of gene functions, if genes encoding kinases evolved particularly fast, and/or those encoding ion transmembrane transporters evolved particularly slow. Both gene groups were found to evolve slower than the rest of genes in the network (median *ω* values for ion transporters, kinases, and the entire network: 0.039, 0.045, and 0.065, respectively; Mann–Whitney tests, *P* = 3.99 × 10^–4^ for ion transporters, *P* = 2.39 × 10^–8^ for kinases), raising the possibility that the higher levels of selective constraint acting at the downstream part of the network would be due to the enrichment of this part of the network in ion transmembrane transporters.

In order to discard this possibility, analyses were repeated excluding the 27 network genes encoding ion transmembrane transporters, with similar results, although not statistically significant in all cases. First, genes are significantly less selectively constrained than their downstream direct targets (paired test, *P* = 0.003 for *ω*; *P* = 2.62 × 10^–4^ for *d*_N_). Second, the 332 non-ion transporter proteins classified as upstream exhibit higher *ω* and *d*_N_ values than the 230 non-ion transporter proteins acting at the downstream part of the network, although the differences are not statistically significant (median *ω* values for upstream and downstream genes: 0.068 and 0.076, respectively; Mann–Whitney test, *P* = 0.181 for *ω*; *P* = 0.203 for *d*_N_). Third, the *ω* and *d*_N_ values correlate better with in-degree (*ρ* = −0.108, *P* = 0.001 for *ω*; *ρ* = −0.109, *P* = 4.72 × 10^–4^ for *d*_N_), than with out-degree (*ρ* = −0.092, *P* = 0.003 for *ω*; *ρ* = −0.092, *P* = 0.003 for *d*_N_), and they exhibit a positive, although non-significant, correlation with *H* (*ρ* = 0.048, *P* = 0.122 for *ω*; *ρ* = 0.051, *P* = 0.104 for *d*_N_). When genes encoding kinases were also excluded from the analysis, the correlation between *H* and rates of evolution reached significance (*ρ* = 0.070, *P* = 0.046 for *ω*; *ρ* = 0.079, *P* = 0.023 for *d*_N_). These results indicate that, although the different functions of upstream and downstream genes may partially contribute to the observed polarity in the levels of selective constraint, this factor does not completely account for the trend.

### Genes acting at the downstream part of the network exhibit higher expression levels and breadths, and encode more highly connected proteins

Levels of selective constraint acting on a gene are affected by a number of factors, including gene expression level (measured, for instance, as the number or concentration of transcripts in the cell) and breadth (the number of tissues in which a gene is expressed) [7,50-52], codon bias [51,53], connectivity of the encoded proteins [14-16], and the number of paralogs [54,55]. Measures of these parameters were obtained from different sources (see Methods). Because the dataset generated by Cui *et al*. [34] focuses on signaling molecules, proteins’ total connectivities were measured as the total number of physical interactors in the whole human protein–protein interaction network [56]. All these factors significantly correlate with *ω* and *d*_N_ in the dataset used (Table 4). Therefore, a putative polarity in the distribution of these factors across the network could potentially account for the observed polarity in the levels of selective constraint. Remarkably, expression breadth and connectivity seem to be the best correlates of the rates of evolution, with both variables exhibiting a similar degree of correlation with *ω* and *d*_N_ (Table 4). This result contrasts with previous observations that connectivity is a relatively weak predictor of rates of evolution (e.g., refs. [5,57]), suggesting that this correlation is particularly strong for genes involved in signal transduction. Furthermore, the correlation between connectivity and rates of evolution remains significant when expression level and breadth, codon bias, and number of paralogs are simultaneously controlled for (partial correlation, *ρ* = −0.183, *P* = 1.67 × 10^–8^ for *ω*; *ρ* = −0.197, *P* = 1.08 × 10^–9^ for *d*_N_). Although genome-level analyses have revealed that the length of the encoded proteins also correlates with *ω* and *d*_N_[7,52], these correlations are not significant in the gene set used (*ρ* = 0.036, *P* = 0.240 for *ω*; *ρ* = 0.024, *P* = 0.431 for *d*_N_), and hence this parameter was not considered.

**Table 4:** Correlates of evolutionary rates

**Variable 1**	** Variable 2**	***ρ***	***P***
*ω*	Expression level	−0.132	2.51×10^–5^***
	Expression breadth	−0.205	4.57×10^–11^***
	ENC	0.163	1.19×10^–7^***
	Connectivity	−0.202	3.35×10^–10^***
	Number of paralogs	−0.116	1.64×10^–4^***
*d*_N_	Expression level	−0.141	6.51×10^–6^***
	Expression breadth	−0.228	1.76×10^–13^***
	ENC	0.002	0.947
	Connectivity	−0.226	1.68×10^–12^***
	Number of paralogs	−0.089	0.004**

**, *P*<0.01, ***, *P*<0.001.

I evaluated whether factors correlating with *ω* and *d*_N_ differ between upstream and downstream genes. The paired test shows that genes tend to exhibit a lower expression breadth (*P* = 0.002), and to encode proteins with a lower connectivity (measured as the number of interactors in the entire human interactome; ref. [56]) (*P* = 8.66 × 10^–18^) than their direct downstream targets (Table 1). The test is only marginally significant for expression level (*P* = 0.082). As expected, both in-degree and out-degree positively correlate with connectivity. The correlation is stronger for in-degree (*ρ* = 0.275, *P* = 5.07 × 10^–18^) than for out-degree (*ρ* = 0.230, *P* = 7.38 × 10^–13^; Table 3), consistent with a higher connectivity in the downstream part of the network. Genes occupying extreme downstream positions in the pathway exhibit higher levels and breadths of expression, and encode more highly connected proteins (Figure 1), than upstream genes, although none of these differences is statistically significant (Table 2). The lack of significance in the Mann–Whitney tests might result from a reduced statistical power resulting from the fact that only a fraction of the genes were used in this analysis (i.e., those occupying extreme upstream or downstream positions), whereas all genes were used in the other two analyses (paired test and correlation analysis).

Although none of the studied factors significantly depends on the upstream/downstream position of genes in all three analyses, these results provide evidence that expression breadth and connectivity, and perhaps expression level, exhibit a polarity across the human signaling network, with downstream genes being more highly and broadly expressed, and encoding more highly connected proteins than upstream genes. Highly and broadly expressed genes [7,50-52], and those encoding highly connected proteins [14-16], tend to be highly constrained. Combined, these trends raise the possibility that the polarity observed in the levels of selective constraint could be the result of the distribution of these factors across the network.

### The lower evolutionary rates of downstream network genes are a byproduct of the distribution of protein connectivity and gene expression level and breadth across the network

I considered whether the observed polarity in the levels of selective constraint across the human signaling network was the result of the distribution of factors correlating with *ω* and *d*_N_. For that purpose, this polarity was evaluated after discounting the putative effects of these factors. Linear regression was used to model the dependence of *ω* and *d*_N_ from each factor separately, and the residuals of the model for each gene (i.e., the difference between the observed and expected values) were used in the paired and the Mann–Whitney tests.

The paired tests show that genes exhibit significantly higher *ω* and *d*_N_ values than their downstream targets even if the effect of expression level, expression breadth, codon bias, or the number of paralogs is discounted, indicating that the difference in the intensity of purifying selection acting on upstream and downstream genes is independent of these factors. However, when the effect of connectivity is discounted, the test yields no significant results (Additional file 1: Table S1).

Genes occupying extreme upstream positions in the network are significantly less constrained than those acting at the downstream part, even when the effect of expression breadth, codon bias, or the number of paralogs is removed, but the difference is not significant when expression level or connectivity are included in the analysis (Additional file 1: Table S2). Given that the relationship between evolutionary rates and their correlates is not necessarily linear in all cases, a complementary nonparametric analysis was performed to assess the differences in *ω* and *d*_N_ values between genes occupying extreme upstream and downstream positions. Partial correlation analysis was used to evaluate the association between upstream/downstream position (encoded as a binary variable; see refs. [58,59]) and rates of evolution while controlling for each of the controlling variables. Similar results were obtained: upstream and downstream genes exhibit significantly higher *ω* and *d*_N_ values when controlling for codon bias or the number of paralogs, but not when controlling for expression level, expression breadth, or connectivity (Additional file 1: Table S3).

Finally, partial correlation analysis was used to evaluate the association between *H* and *ω* and *d*_N_ while controlling separately for each factor. Both correlations are significant when codon bias or the number of paralogs are used as controlling variables, but not when expression level, expression breadth, or connectivity are included in the analysis (Additional file 1: Table S4).

Therefore, the differences in the *ω* and *d*_N_ values of upstream and downstream network genes disappear when the effect of expression level, expression breadth, or connectivity, are removed. This indicates that these factors account for the higher levels of selective constraint acting on the downstream genes of the human signal transduction network. In particular, when connectivities are controlled for, *ω* and *d*_N_ values are not significantly different for upstream and downstream genes, regardless of the method used (Additional file 1: Tables S1–S4), indicating that this factor may be the main responsible for the polarity in the levels of selective constraint. When the effects of expression level or breadth are accounted for, the differences in *ω* and *d*_N_ between upstream and downstream genes vanish for only one or two of the three methods used, indicating that these factors may contribute to the selective constraint polarity to a lesser extent.

### A case study: Natural selection upstream and downstream of the mammalian Ras signaling pathway

So far, the *Drosophila* Ras pathway is the only signal transduction pathway for which higher levels of selective constraint have been described in genes acting at the upstream part [37]. The Ras pathway is activated by diverse extracellular stimuli, including growth factors, cytokines and hormones. Activation of receptor tyrosine kinases (RTKs, such as EGFR) triggers a cascade of signaling events (including a cascade of protein phosphorylations) that ultimately promote cell proliferation, differentiation, migration and survival (for review, see ref. [60]). The architecture of this pathway has been characterized in detail in a number of organisms, including *Drosophila* and mammals, revealing a highly conserved structure and function across metazoans. Riley *et al*. [37] examined the patterns of molecular evolution of six of the genes involved in the *Drosophila* Ras pathway. For that purpose, they surveyed the levels and patterns of polymorphism in *Drosophila melanogaster*, and divergence with respect to the sister species *D. simulans*. They found that three proteins acting at the upstream part of the pathway (Drk, Ras and Raf) were identical in both species, and exhibited no or just a few rare replacement polymorphisms within *D. melanogaster*. On the contrary, the three downstream proteins included in their analysis (Dsor1, Ksr and Corkscrew) displayed a number of fixed differences between the two species, as well as some replacement polymorphisms in *D. melanogaster*. Consistent results were obtained from the *ω* values (ranging 0.000–0.003 for the upstream genes, and 0.006–0.110 for the downstream ones), pointing out to higher levels of purifying selection acting at the upstream part of the pathway. A proposed explanation for this observation was that, owing to their highly hierarchical position, proteins acting at the upstream part of the pathway would act as key control points, greatly influencing the activity of downstream proteins, whereas downstream proteins would act as modulators of the output signal [37].

**Figure 2:**
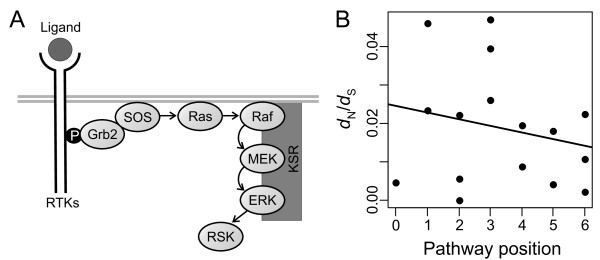
**Structure of the mammalianRas pathway (A) anddistribution of selective pressuresacross the upstream/downstream pathwayaxis (B).** (**A**) Upon binding to mitogenic ligands (e.g. EGF), receptor tyrosine kinases (RTKs, e.g. EGFR), are able to recruit Grb2 to the cell membrane. Grb2 binds to SOS proteins, thus promoting their membrane location. Once in the membrane, SOS proteins act as guanine exchange factors of Ras proteins, thereby promoting Ras’ activation. Activated forms of Ras promote the recruitment of Raf proteins to the membrane, which in turn phosphorylate MEK proteins. Activated MEK proteins then phosphorylate ERK proteins. The required molecular interactions for these phosphorylation events are facilitated by interaction with the scaffold proteins KSR. Finally, ERK proteins phosphorylate RSK proteins, which in turn activate ribosomal protein S6 and a number of transcription factors, thus promoting cell proliferation, differentiation, migration and survival, and modulating cellular metabolism. (**B**) Correlation between the position of genes in the pathway (defined as the number of steps required for the signal transduction between RTKs to each of the genes in the pathway) and their rates of evolution. See Additional file 1: Table S5 for a full list of the core pathway genes and their rates of evolution.

The current wealth of genomic data provides an excellent opportunity to study the patterns of molecular evolution of all the core components of the Ras pathway in other taxonomic groups. Hence, I considered whether levels of selective constraint acting on the mammalian Ras pathway genes exhibit a similar distribution across the pathway to that observed in *Drosophila*. Most of the afore-mentioned *Drosophila* pathway genes have multiple orthologs in mammalian genomes known to participate in the Ras pathway (e.g., ref. [60]). For each such mammalian gene, the *ω* value was estimated from comparison of the human-mouse pair of orthologs (see Additional file 1: Table S5). Genes encoding the mammalian proteins SOS1, SOS2, ERK1, ERK2, and RSK1–3, which are also key components of the pathway [60], were also included in the current analysis (see Figure 2A for a diagram and description of the pathway). Contrary to previously suggested in *Drosophila*[37], genes acting at the upstream part of the mammalian Ras pathway (those encoding the Grb2, SOS1, SOS2, H-Ras, K-Ras, N-Ras, A-Raf, B-Raf, and C-Raf proteins) were not found to exhibit lower rates of evolution than those acting at the downstream part (the rest of genes listed in Additional file 1: Table S5). If anything, the opposite seemed to be the case (median *ω* for upstream and downstream genes: 0.023 and 0.018, respectively), although the differences between both groups were not significant (Mann–Whitney test, *P* = 0.503). Consistently, *ω* values were found to exhibit a negative, although non-significant, correlation with the position of genes along the upstream/downstream axis of the pathway (*ρ* = −0.195, *P* = 0.470; Figure 2B). For this analysis, pathway position was defined as the number of steps required to transduce the signal from the most upstream element (Grb2, position 0), to each of the pathway components (with proteins RSK1–3 sharing position 6, the most downstream position of the core pathway; Figure 2A; Additional file 1: Table S5).

Neither expression levels, nor expression breadths or connectivities significantly correlate with pathway position (expression level: *ρ* = 0.107, *P* = 0.693; expression breadth: *ρ* = 0.116, *P* = 0.670; connectivity: *ρ* = −0.134, *P* = 0.620) or with *ω* values (expression level: *ρ* = 0.203, *P* = 0.450; expression breadth: *ρ* = 0.123, *P* = 0.649; connectivity: *ρ* = −0.174, *P* = 0.519). It should be noted, however, that microarray-based gene expression data, such as the one used here [61,62], is subject to high levels of measurement noise. More accurate measures of expression are available for some of the proteins involved in the Ras pathway, and indeed it has been noticed that downstream proteins are present in higher concentrations in the cell (e.g., see ref. [38] and references therein), thus leaving open the possibility that the slightly higher levels of selective constraint observed in the downstream part of the pathway (Figure 2; Additional file 1: Table S5) may result from these genes being expressed at higher levels.

In any case, these results indicate that, contrary to previously suggested in *Drosophila*[37], genes acting at the upstream part of the mammalian Ras pathway are not subject to higher levels of selective constraint than those acting at the downstream part. If anything, the opposite might be the case, although the trend is not statistically significant. This observation is in agreement with the general trend described here for the whole human signal transduction network.

## Discussion

In summary, results presented here demonstrate that genes acting at the upstream part of the human signal transduction network clearly do not evolve under stronger selective pressures (Tables 1– 3; Figure 1). This finding seems striking, as these proteins, owing to their higher hierarchical position, may be capable of regulating the activity of a higher number of downstream proteins. Therefore, mutations in the genes encoding upstream network proteins would be expected to have more pleiotropic effects, and hence these genes would be expected to evolve under stronger purifying selection. In contrast with these expectations, a number of analyses that capture different aspects of the hierarchical position of genes in the signaling network found no evidence for upstream genes being subject to higher levels of selective constraint. On the contrary, upstream network genes are less selectively constrained than downstream genes, which appears to be explained by downstream genes performing certain functions, being more highly and broadly expressed and, in particular, encoding more highly connected proteins.

Protein connectivity, gene expression breadth and, to a lesser extent, expression level, exhibit a polarity across the human signaling network, with downstream genes progressively encoding proteins that are more connected, and being expressed in a higher number of tissues and at higher levels (Tables 1,3; Figure 1). The higher levels of expression of downstream genes might facilitate the amplification of signals through their progression throughout signaling cascades, which requires higher protein abundances for the downstream elements of the pathway (e.g., ref. [38]). Additionally, the higher connectivity of downstream genes is consistent with previous observations that hubs (i.e., highly connected proteins) tend to be tightly regulated, exhibiting higher numbers of phosphorylation sites and higher mRNA decay rates [57]. The distribution of these factors across the network, combined with the fact that highly and broadly expressed genes [7,50-52], and those encoding highly connected proteins in the protein–protein interaction network [14-16], tend to evolve under stronger purifying selection, accounts for the higher levels of selective constraint observed in genes acting at the downstream part of the network. In particular, when the effect of connectivity is factored out, the differences in *ω* and *d*_N_ values between upstream and downstream genes are no longer significant, regardless of the method used (Additional file 1: Tables S1–S4), pointing to this factor as the main responsible for the higher levels of selective constraint acting on the downstream network genes. When the effects of expression level and breadth are removed, the differences in the levels of selective constraint observed between upstream and downstream genes also vanish for some of the tests (Additional file 1: Tables S1–S4), suggesting that these factors also contribute to the tendency. Likewise, protein functions may also play a role, as the trend also vanishes for some tests when ion transmembrane transporters (which are slow-evolving and are enriched in the downstream part of the network) are removed from the analyses. In any case, taken together these observations indicate that the upstream/downstream position of proteins in the human signal transduction network does not have a direct effect on their rates of evolution.

The global trends observed in the entire human signal transduction network (i.e., downstream genes being more selectively constrained owing to their higher connectivity, expression level and breath) mirror those previously observed in some particular pathways. In both the *Caenorhabditis* insulin/TOR signaling pathway and the yeast HOG signaling pathway, downstream genes evolve under stronger levels of selective constraint, probably as a result of their higher levels of expression and codon bias (which is often used as a proxy for levels of expression), respectively [42,43]. It should be noted, however, that the general trends observed across the whole network do not necessarily imply that each particular signaling pathway must exhibit the same trend. For instance, in the *Drosophila* and vertebrate insulin/TOR pathways, downstream genes evolve under stronger purifying selection, even when the putative effect of several factors that correlate with levels of selective constraint is discounted [39-41,44]. Another example that does not seem to conform to the general trend described here is the *Drosophila* Ras pathway [37], whose upstream genes appear to be the most constrained, despite these genes being the ones expressed at lower levels [38] (however, observations in the mammalian Ras pathway do not point in the same direction; see below, Figure 2 and Additional file 1: Table S5). Therefore, despite the overall trends described here, the particular structure and function of each individual pathway may result in different distributions of the impact of evolutionary forces. For instance, pathways whose upstream genes, for some reason, present higher connectivities, and/or levels or breadths of expression, would probably be more selective constrained in the upstream part. It should also be noted that factors other than the ones considered here may also affect the distribution of levels of selective constraint across functional pathways and networks. In particular, enzymes whose kinetic characteristics exert a high degree of influence over the entire network (and hence, on the phenotype) are expected to be more selectively constrained than those exerting little influence on the network dynamics [31,63-65]. The degree to which a particular enzyme can influence the behavior of the entire network is, however, difficult to determine from currently available data.

So far, the *Drosophila* Ras pathway is, to the best of the author’s knowledge, the only signal transduction pathway whose upstream genes appear to be the ones evolving under higher levels of purifying selection [37]. However, due to the limited availability of genomic data at that time, this observation was based on the analysis of a small set of genes and the comparison of two closely related species (*D. melanogaster* and its sister species *D. simulans*, which are thought to have diverged 2–6 million years ago [66,67]). Results reported here for all core components of the mammalian Ras pathway, and based on human-mouse comparisons (which probably diverged ~75 million years ago; e.g., ref. [68]), do not point in the same direction: upstream genes do not exhibit lower rates of evolution than those acting at the downstream part of the pathway. If anything, the opposite might be the case (i.e., downstream genes exhibit slightly lower rates of evolution, although no significant differences are observed between upstream and downstream genes; Figure 2; Additional file 1: Table S5). In any case, this observation lends further support to the finding that genes occupying highly hierarchical positions in the human signal transduction network are not subject to higher levels of selective constraint than those acting at the downstream part.

The observation that genes acting at the upstream part of the human signal transduction network are not subject to higher levels of selective constraint sharply contrasts with the trends generally observed in biosynthetic pathways. These pathways consist of a series of enzymes that share metabolites, with the product of a given enzyme being usually the substrate of the following enzyme (or enzymes) in the pathway. In such pathways, metabolites can often take different alternative routes, which end up in the biosynthesis of different end products. In a growing number of biosynthetic pathways, including the plant anthocyanin [28,69,70], isoprene [25], terpenoid [26], and carotenoid pathways [27,71], the *Bombyx* melanin pathway [30], and the mammalian dopamine pathway [29], levels of selective constraint correlate with the position of genes along the upstream/downstream pathway axis, with upstream genes being always the most constrained. Two plausible scenarios might explain such a distribution of selective constraints in biosynthetic pathways. First, as a result of their branching topology, these pathways often exhibit a hierarchical structure, with upstream genes being necessary for the biosynthesis of a higher number of end products than downstream genes; therefore, mutations in genes acting at the upstream part may have higher pleiotropic effects [28]. Second, upstream enzymes in biosynthetic pathways probably often exert a higher control over the flux of metabolites throughout the pathway [31]; thus, the overall pathway function (and, hence, the associated phenotypes) may more strongly depend on the function of these enzymes. Both models predict a higher relevance for pathway function of upstream genes, and hence a higher strength of natural selection acting on these genes. Similar trends have been observed in transcriptional regulatory networks. First, genes occupying higher hierarchical positions in these networks are likely to be essential, reflecting their key importance for organism’s fitness [32]. Second, simulation analyses of transcriptional regulatory networks have shown that genes exerting a high degree of control over other genes, or those that are less regulated by other genes, are expected to be more strongly affected by selection [33].

Therefore, results presented here imply that the distribution of evolutionary forces across the upstream and downstream parts of the signal transduction network remarkably differs from that observed in biosynthetic and transcriptional regulatory pathways. In biosynthetic and transcriptional regulatory pathways, upstream genes evolve under stronger levels of selective constraint, whereas this is not the case for the human signal transduction network. This suggests that genes occupying higher hierarchical positions are not more relevant for the function of the signaling network, or to the organism’s fitness, than downstream genes. These different patterns of evolution may reflect fundamental differences in the function and organization of the different kinds of pathways.

As signaling networks become better understood, it becomes more apparent that signal transduction does not take place in the context of independent linear pathways in which each protein is exclusively controlled by the preceding protein in the cascade. This classical model is being progressively substituted by a much more integrative model of signal transduction, in which a considerable amount of cross-talk takes place between pathways. As a result, pathways would cooperate to integrate signals, and to make decisions about the responses in a decentralized manner, thus conferring robustness to the system [72,73]. This kind of organization may allow the network to accommodate modifications in its upstream genes. For instance, even if an upstream gene undergoes disruptive mutation, its downstream targets might still be activated by alternative routes that do not involve the disrupted element. The observation that genes occupying more hierarchical positions in the signaling network are not subject to stronger selective forces is consistent with this emerging integrative model. Although somewhat speculative, this observation would be compatible with the organization of the signal transduction network being less hierarchical than that of biosynthetic and transcriptional regulatory networks. In the future, a deeper understanding of the differences between the nature of biosynthetic, transcriptional regulatory, and signaling pathways may shed more light on their different patterns of evolution.

## Conclusions

Surprisingly, genes occupying the higher hierarchical positions of the human signal transduction network are not subject to stronger levels of purifying selection, suggesting that they are not more important for the function of the network and the fitness of the organism than genes occupying the lower hierarchical positions. This observation sharply contrasts with the patterns observed in metabolic and transcriptional regulatory pathways and networks, in which upstream genes are generally the most selectively constrained. These contrasting patterns of evolution might reflect fundamental differences in the function and organization of signaling and biosynthetic and transcriptional regulatory networks. In any case, results presented here broaden our knowledge on how natural selection distributes across molecular networks.

## Methods

### Human signal transduction network

The human directed signaling network described in ref. [34] was used. After eliminating metabolites, proteins that could not be mapped to Ensembl IDs, proteins encoded by genes without a 1:1 ortholog in the mouse genome (see below), and undirected interactions (i.e., those for which the directionality of signal transduction is not available), the dataset contained 2436 activating or inhibitory interactions connecting 1049 human proteins.

For each human protein, a number of measures of its hierarchical position in the network were computed: in-degree and out-degree (number of upcoming and outgoing edges in the network, respectively), and the total number of proteins that lay downstream of the protein of interest (termed *H*). Proteins with high out-degrees (i.e., modulating a high number of other proteins), low in-degrees (i.e., modulated by a few or no other proteins), and/or high *H* values (i.e., with a high number of proteins acting downstream of it) can be considered to occupy high hierarchical positions in the network. *H* was computed as follows: first, a list of proteins containing all downstream direct targets of the protein of interest was generated; second, all direct downstream targets of these proteins were incorporated to the list. The second step was iterated until all proteins acting downstream of the protein of interest were found (i.e., until the number of elements in the list remained stable).

### Impact of natural selection

All human and mouse protein and CDS sequences were obtained from the Ensembl database, release 62 [74]. For genes encoding multiple transcripts owing to alternative splicing, the variant encoding the longest protein (i.e., the “canonical transcript”) was used. For each protein in the human signaling network, the 1:1 mouse ortholog was identified using a best reciprocal BLAST approach (using BLASTP and an *E*-value cut-off of 10^–10^). Pairs of orthologous amino acid sequences were aligned using ProbCons 1.12 [75], and the resulting alignments were used to guide the alignment of the corresponding CDSs.

For each pair of orthologous sequences, the impact of natural selection was characterized from the nonsynonymous (*d*_N_) to synonymous (*d*_S_) divergence ratio (*ω* = *d*_N_/*d*_S_). Estimates of *ω*, *d*_N_ and *d*_S_ were obtained using the program codeml from the package PAML 4.4 [76] (under the M0 model). The action of positive selection was inferred using the M7 *vs.* M8 test [45]. Model M7 assumes that codons’ *ω* values range between 0 and 1, following a beta distribution, whereas model M8 allows for an extra class of codons with *ω* > 1. The likelihood of both models is compared using the likelihood ratio test [77], assuming that twice the difference between the log-likelihoods of both nested models (2Δ*ℓ*) follows a *χ*^2^ distribution with 2 degrees of freedom. If model M8 significantly better fits the data, positive selection is invoked. *P*-values for this test were corrected for multiple testing using the false discovery rate approach [78].

### Statistical analyses

Three different tests were carried out to determine whether upstream and downstream genes in the network evolve under different levels of selective constraint. First, for each gene with direct downstream targets (i.e., out-degree > 0), its *ω* (and *d*_N_) value was compared with the central values of its direct downstream targets using the paired sign test. If the upstream protein regulated an odd number of downstream proteins, the median of the corresponding *ω* values was used. If the number of downstream targets was even, one of the two central values was chosen at random. This approach was adopted to avoid using the average of the central values, which can be problematic if the distribution of the variable under study (*ω* in this case) is asymmetric. Second, the Mann–Whitney *U* test was used to compare the *ω* and *d*_N_ values for genes encoding proteins with extreme upstream and downstream positions in the network. Proteins were considered to act in the upstream part of the network if they modulate other proteins (out-degree > 0) and are not modulated by any other protein (in-degree = 0), or in the downstream part of the network if out-degree = 0 and in-degree > 0. Third, I evaluated whether the *ω* and *d*_N_ values correlate with measures of hierarchical position in the network (in-degree, out-degree, and *H*) using the Spearman’s rank correlation coefficient (*ρ*). These methods were likewise applied to evaluate whether upstream and downstream genes differ in terms of a number of factors that are known to correlate with the levels of selective constraint: gene expression level and breadth, codon usage bias, connectivity, and number of paralogs (see below).

Furthermore, I considered whether the differences in *ω* and *d*_N_ observed between upstream and downstream genes were attributable to the distribution of their correlates across the network. For the first two methods (paired sign and Mann–Whitney tests), linear regression was used to model the dependence of *ω* and *d*_N_ from each factor. Then, the residuals of the model for each gene (i.e., the difference between the observed and predicted values) were used in the analyses. Additionally, partial correlation analysis was used to evaluate the correlation between rates of evolution and upstream/downstream location while controlling for each variable. For that purpose, upstream/downstream location was encoded as a binary variable (see refs. [58,59]). The Spearman’s rank correlation test, when applied to a continuous and a binary variable, is equivalent to the Mann–Whitney test. Finally, partial correlation analysis was also used to evaluate the correlation between evolutionary rates and the measures of hierarchical position (in-degree, out-degree and *H*) while controlling for each of the controlling variables.

### Correlates of selective constraint levels

For each human gene, estimates for a number of factors that correlate with *ω* and *d*_N_ were obtained from different sources:

**• Gene expression level andbreadth:** Human gene expression data was retrieved from the BioGPS portal [61,62] (U133A/GNF1H dataset gcRMA-normalized). Probes were matched to genes through Ensembl’s BioMart [79] (for the U133A dataset) or the annotation file provided in the BioGPS portal (for the GNF1H dataset). For each probe and tissue, values were averaged across both replicates. For each probe, expression level was computed as the average across a subset of 25 nonredundant, adult noncancerous tissues (as in ref. [40]). For genes matching more than one probe, the one with the highest average across the 25 selected tissues was used. Expression breadth for each gene was calculated as the number of tissues (out of the 25 selected ones) in which the gene is expressed above the median across all tissues and genes.

**• Codon bias:** The effective number of codons (ENC; ref. [80]) of each gene was computed using the software CodonW 1.4.2 ( http://codonw.sourceforge.net/). For each pair of human-mouse orthologs, ENC values were averaged.

**• Number of paralogs:** For each human gene, a list of human paralogs was obtained from Ensembl’s BioMart.

**• Protein connectivity:** The whole human protein–protein interaction network was retrieved from the BioGRID database version 3.0.67 [56]. Only physical interactions between human proteins were used. For each protein, connectivity was computed as the number of proteins with which it physically interacts. Genes not represented in the database were assigned missing values.

**• Subcellular location:** For each gene, a list of the Gene Ontology [81] terms to which it is associated was obtained from Ensembl’s BioMart. Genes were considered to act in the extracellular space (105 genes), in the plasma membrane (381), cytoplasm (570) or in the nucleus (455) if associated to the terms “extracellular region”, “plasma membrane”, “cytoplasm” or “nucleus”, respectively. Among these, 34, 130, 125 and 121 act exclusively in the extracellular compartment, the plasma membrane, the cytoplasm and the nucleus, respectively.

## Competing interests

The author declares that he has no competing interests.

## Supplementary Material

Additional file 1 **A single PDF file containing supplementary Tables S1–S5.** Table S1 lists the results of the paired tests comparing the evolutionary rates of each gene with those of its direct downstream targets while controlling for a number of correlates of rates of evolution. Table S2 lists the Mann–Whitney tests comparing the evolutionary rates of genes occupying extreme upstream and downstream positions while controlling for a number of correlates of rates of evolution. Table S3 lists the partial correlation analyses contrasting the association between upstream/downstream position of genes and their rates of evolution while controlling for a number of correlates of rates of evolution. Table S4 lists the partial correlation analyses contrasting the association between rates of evolution and measures of hierarchical position while controlling for a number of correlates of rates of evolution. Table S5 lists the *ω* values for genes involved in the mammalian Ras signaling pathway. Click here for file
