# Supplementary material for: The relationship between the hierarchical position of proteins in the human signal transduction network and their rate of evolution
Source: BMC Evol Biol. 2012 Sep 28;12:192. doi: 10.1186/1471-2148-12-192 (PMC3527147; doi:10.1186/1471-2148-12-192)
Supplement: Additional file 1 — A single PDF file containing supplementary Tables S1–S5. Table S1 lists the results of the paired tests comparing the evolutionary rates of each gene with those of its direct downstream targets while controlling for a number of correlates of rates of evolution. Table S2 lists the Mann–Whitney tests comparing the evolutionary rates of genes occupying extreme upstream and downstream positions while controlling for a number of correlates of rates of evolution. Table S3 lists the partial correlation analyses contrasting the association between upstream/downstream position of genes and their rates of evolution while controlling for a number of correlates of rates of evolution. Table S4 lists the partial correlation analyses contrasting the association between rates of evolution and measures of hierarchical position while controlling for a number of correlates of rates of evolution. Table S5 lists the ω values for genes involved in the mammalian Ras signaling pathway. [file 1471-2148-12-192-S1.pdf]

SUPPLEMENTARY MATERIAL

Table S1. Paired tests comparing ω and d_N values of upstream genes with their downstream targets controlling for a number of variables

Parameter	Controlling variable	n[a]	Upstream > Downstream[b]	Upstream < Downstream[c]	P
ω	Expression level	763	418	345	0.009**
	Expression breadth	763	418	344	0.008**
	ENC	800	435	365	0.015*
	Connectivity	709	360	349	0.707
	Number of paralogs	800	442	358	0.003**
d_N	Expression level	763	419	344	0.007**
	Expression breadth	763	429	331	4.34×10^{-4}***
	ENC	800	447	353	0.001**
	Connectivity	709	351	358	0.822
	Number of paralogs	800	452	348	2.71×10^{-4}***

*, $P < 0.05$; **, $P < 0.01$; ***, $P < 0.001$.
[a]Number of genes with at least one direct downstream target (out-degree > 0). Only genes with available information for the controlled factor were considered.
[b]Number of genes with a higher value than the central value of its downstream targets.
[c]Number of genes with a lower value than the central value of its downstream targets.

Table S2. Comparison of genes occupying extreme upstream and downstream positions controlling for a number of variables

Parameter	Controlling variable	Upstream			Downstream			P
		n	Median	Average	n	Median	Average	
ω	Expression level	322	–0.0243	0.0105	243	–0.0350	0.0000	0.098
	Expression breadth	322	–0.0225	0.0095	243	–0.0348	–0.0008	0.048*
	ENC	333	–0.0200	0.0120	249	–0.0349	–0.0018	0.041*
	Connectivity	293	–0.0302	0.0087	225	–0.0378	–0.0001	0.099
	Number of paralogs	333	–0.0227	0.0119	249	–0.0349	–0.0017	0.043*
d_N	Expression level	322	–0.0182	0.0073	243	–0.0254	–0.0018	0.110
	Expression breadth	322	–0.0168	0.0066	243	–0.0225	–0.0025	0.035*
	ENC	333	–0.0165	0.0080	249	–0.0251	–0.0026	0.049*
	Connectivity	293	–0.0225	0.0049	225	–0.0266	–0.0018	0.172
	Number of paralogs	333	–0.0168	0.0079	249	–0.0249	–0.0025	0.048*

P-values were obtained from the Mann-Whitney *U* test. *, $P < 0.05$.

Table S3. Partial correlations between evolutionary rates and extreme upstream/downstream location

Variable 1	Controlling variable	*n*	*ρ*	*P*
ω	Expression level	565	0.071	0.092
	Expression breadth	565	0.075	0.075
	ENC	582	0.091	0.028*
	Connectivity	518	0.057	0.191
	Number of paralogs	582	0.086	0.037*
d_N	Expression level	565	0.071	0.093
	Expression breadth	565	0.075	0.075
	ENC	582	0.082	0.048*
	Connectivity	518	0.041	0.348
	Number of paralogs	582	0.081	0.049*

Upstream/downstream location was encoded as a binary variable (1 for upstream, 0 for downstream). *, $P < 0.05$.

Table S4. Partial correlations between parameters of interest and measures of hierarchical positions of genes in the network controlling for a number of variables

Variable 1	Variable 2	Controlling variable	*n*	*ρ*	*P*
ω	in-degree	Expression level	1016	–0.112	3.36×10^{-4}***
		Expression breadth	1016	–0.105	0.001***
		ENC	1049	–0.119	1.04×10^{-4}***
		Connectivity	951	–0.058	0.073
		Number of paralogs	1049	–0.114	2.10×10^{-4}***
	out-degree	Expression level	1016	–0.082	0.009**
		Expression breadth	1016	–0.069	0.028*
		ENC	1049	–0.068	0.028*
		Connectivity	951	–0.036	0.261
		Number of paralogs	1049	–0.065	0.034*
	H	Expression level	1016	0.054	0.085
		Expression breadth	1016	0.051	0.103
		ENC	1049	0.071	0.021*
		Connectivity	951	0.052	0.106
		Number of paralogs	1049	0.070	0.024*
d_N	in-degree	Expression level	1016	–0.114	2.64×10^{-4}***
		Expression breadth	1016	–0.106	0.001***
		ENC	1049	–0.117	1.37×10^{-4}***
		Connectivity	951	–0.045	0.170
		Number of paralogs	1049	–0.116	1.68×10^{-4}***
	out-degree	Expression level	1016	–0.080	0.011*
		Expression breadth	1016	–0.066	0.036*
		ENC	1049	–0.072	0.019*
		Connectivity	951	–0.031	0.332
		Number of paralogs	1049	–0.068	0.027*
	H	Expression level	1016	0.058	0.064
		Expression breadth	1016	0.055	0.080
		ENC	1049	0.067	0.030*
		Connectivity	951	0.050	0.127
		Number of paralogs	1049	0.069	0.025*

*, $P < 0.05$; **, $P < 0.01$; ***, $P < 0.001$.

Table S5. Evolutionary rates in the mammalian Ras pathway genes

***Drosophila* gene**	**Human orthologs[a]**	**Human protein**	**Pathway position[b]**	**ω[c]**
drk	*GRB2*	Grb2	0	0.0047
Sos	*SOS1*	SOS1	1	0.0234
	SOS2	SOS2	1	0.0461
Ras85D	*HRAS*	H-Ras	2	0.0001
	KRAS	K-Ras	2	0.0222
	NRAS	N-Ras	2	0.0058
phl	*ARAF*	A-Raf	3	0.0472
	BRAF	B-Raf	3	0.0396
	RAF1	C-Raf	3	0.0262
Dsor1	*MAP2K1*	MEK1	4	0.0088
	MAP2K2	MEK2	4	0.0196
rl	*MAPK1*	ERK2	5	0.0043
	MAPK3	ERK1	5	0.0181
S6kII	*RPS6KA1*	RSK1	6	0.0108
	RPS6KA2	RSK3	6	0.0225
	RPS6KA3	RSK2	6	0.0022
ksr	*KSR1*	KSR1	–	0.0645
	KSR2	KSR2	–	0.0295
csw	*PTPN6*	SHP1	–	0.0308
	PTPN11	SHP2	–	0.0029

[a]Human genes known to be involved in the Ras pathway.
[b]Pathway position is defined as the number of steps required to transduce the signal from Grb2 to each of the pathway components. Genes encoding the SHP and KSR proteins were not assigned a precise pathway position, as they do not directly participate in the core signaling cascade (see Fig. 2A and refs. 60 and 82).
[c]Nonsynonymous to synonymous divergence ratio (ω = d_N/d_S) estimated from the comparison of human-mouse pairs of orthologs.
